# The complete chloroplast genome sequence of *Firmina danxiaensis*

**DOI:** 10.1080/23802359.2020.1715294

**Published:** 2020-01-27

**Authors:** Xiao-Yu Lin, Zhi-Hui Chen, Yun-Yuan Yang, She-Qin Mo, Dong-Lin Li

**Affiliations:** College of Ying-Tong Agricultural Science and Engineering, Shaoguan University, Shaoguan, China

**Keywords:** *Firmina danxiaensis*, Firmina, chloroplast genome, phylogenomic tree

## Abstract

The complete chloroplast genome of *Firmina danxiaensis*, an important deciduous tree, was identified and sequenced in this study. The genome size is 161,205 bp, the GC content is 36.88%. A total of 129 genes were identified, including 84 protein-coding genes, 8 rRNA genes, and 37 tRNA genes. 10 plastome accessions from Sterculiaceae were selected to assess the phylogenetic placement of genus and the result showed that *F. danxiaensis* is most closely related to *F. simplex*.

Danxia Mountain is located in a special geographical location of the Nanling Mountains, which is the natural boundary between the north and south of vegetation (He et al. 1991). A large number of rare and endemic species have been preserved in the area, such as *Firmina danxiaensis*, *Chiritopsis danxiaensis* and *Spiradiclis danxiashanensis*. *F. danxiaensis*, Sterculiaceae, is a small deciduous tree characterized by beautiful tree shape, flower colors, and golden autumn leaves (Xu et al. 1987). It is an endemic species in Guangdong Province, and only distributed in Danxia Mountain and Cangshi Village in Shaoguan. Additionally, it was listed as a critically dangerous (CR) grade in the assessment of the first volume of the Chinese Red List of Species (Wang and Xie 2004). Previous research reports on *F. danxiaensis* mainly focus on the analysis of the geographical flora of its community (Luo et al. 2015), the spatial distribution of micro-geomorphic environmental features (Ouyang et al. 2017), and genetic diversity (Fan et al. 2013; Chen et al. 2014), no chloroplast genome resource is available so far for this important tree. In this study, the first complete chloroplast genome of *F. danxiaensis* is reported.

The fresh leaves of *F. danxiaensis* was collected from Xiafu village (25°0′04″N, 113°41′44″E) in Shaoguan, Guangdong, China, and the Voucher specimens were deposited in the Herbarium of Shaoguan university, the accession number is Li-201905. Total genomic DNA was extracted from the fresh mature leaves using the Plants Genomic DNA Kit (DP305, Tiangen Biotech Co., Ltd., Beijing, China). The plastome sequences was generated using Illumina HiSeq 2500 platform (Illumina Lnc., San Diego, CA, USA). In total, 6.5 Gb raw reads were obtained. The filtered reads were assembled with the program NOVOPlasty 3.1 (Dierckxsens et al. 2017) with a part of *rbcL* gene of *Antiaris toxicaria* (NC 042884), and then the sequence of *F. danxiaensis* was annotated using DOGMA (Wyman et al. 2004). The annotated sequence was submitted to NCBI, the accession number is MN720649.

The full length of *F. danxiaensis* chloroplast genome was 161,205 bp. It is made up of a large single-copy region (LSC with 90,114 bp), a small single-copy region (SSC with 20,057 bp) and two inverted repeat regions (IRs with 25,517 bp). Total GC content is 36.88%. A total of 129 genes are successfully annotated, including 84 protein-coding genes, 37 tRNA genes, and 8 rRNA genes. The tRNA genes are distributed throughout the whole genome with 21 in the LSC, 3 in the SSC, and 9 in the IR regions, while rRNAs are only situated in the IR regions.

To further investigate the phylogenetic position of *F. danxiaensis* in Sterculiaceae family, 10 of complete chloroplast genomes in Sterculiaceae family was download from NCBI, and then the maximum-likelihood (ML) phylogenetic tree was generated by MEGA 7.0 (Kumar et al. 2016), with *Heritiera eleta* as outgroup. The results in Figure 1 shows that *F. danxiaensis* is closed to *F. simplex*. This newly reported chloroplast genome will provide valuable information for genetic evolution and molecular breading studies of *Firmina*.

**Figure 1.**
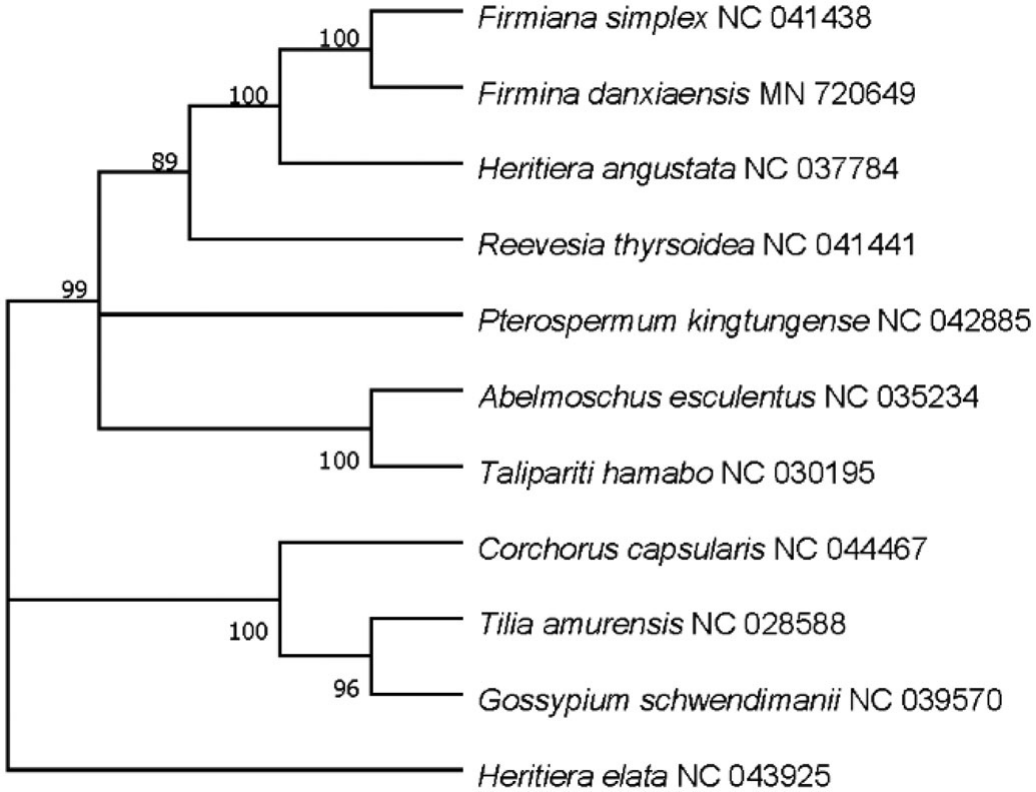
Maximum likelihood tree based on the sequences of ten complete chloroplast genomes. Numbers in the nodes were bootstrap values from 1000 replicates. Scale in substitutions per site.
